# A Multivariate Metabolomics Method for Estimating Platelet Mitochondrial Oxygen Consumption Rates in Patients with Sepsis

**DOI:** 10.3390/metabo10040139

**Published:** 2020-04-02

**Authors:** Marc R. McCann, Cora E. McHugh, Maggie Kirby, Theodore S. Jennaro, Alan E. Jones, Kathleen A. Stringer, Michael A. Puskarich

**Affiliations:** 1 The NMR Metabolomics Laboratory, Department of Clinical Pharmacy, College of Pharmacy, University of Michigan, Ann Arbor, MI 48109, USA; mrmccann@umich.edu (M.R.M.); mchughce@med.umich.edu (C.E.M.); tjennaro@med.umich.edu (T.S.J.); stringek@umich.edu (K.A.S.); 2 Department of Emergency Medicine, University of Mississippi Medical Center, Jackson, MS 39216, USA; maggie.kirby@vumc.org (M.K.); aejones@umc.edu (A.E.J.); 3 Division of Pulmonary and Critical Care Medicine, Department of Medicine, School of Medicine, University of Michigan, Ann Arbor, MI 48109, USA; 4 Michigan Center for Integrative Research in Critical Care, University of Michigan, Ann Arbor, MI 48109, USA; 5 Department of Emergency Medicine, University of Minnesota, Minneapolis, MN 55455, USA

**Keywords:** nuclear magnetic resonance spectroscopy, acetylcarnitine, bioenergetics, mitochondrial function, mitochondrial respiration, mitochondria, metabolism

## Abstract

Background: Sepsis-induced alterations in mitochondrial function contribute to organ dysfunction and mortality. Measuring mitochondrial function in vital organs is neither feasible nor practical, highlighting the need for non-invasive approaches. Mitochondrial function may be reflected in the concentrations of metabolites found in platelets and whole blood (WB) samples. We proposed to use these as alternates to indirectly estimate platelet mitochondrial oxygen consumption rate (mOCR) in sepsis patients. Methods: We determined the relationships between platelet mOCR and metabolites in both platelets and WB, as measured by quantitative ^1^H-NMR metabolomics. The associations were identified by building multiple linear regression models with stepwise forward-backward variable selection. We considered the models to be significant with an ANOVA test (*p*-value ≤ 0.05) and a positive predicted-R^2^. Results: The differences in adjusted-R^2^ and ANOVA *p*-values (platelet adj-R^2^: 0.836 (0.0003), 0.711 (0.0004) vs. WB adj-R^2^: 0.428 (0.0079)) from the significant models indicate the platelet models were more associated with platelet mOCR. Conclusions: Our data suggest there are groups of metabolites in WB (leucine, acetylcarnitine) and platelets (creatine, ADP, glucose, taurine) that are associated with platelet mOCR. Thus, WB and platelet metabolites could be used to estimate platelet mOCR.

## 1. Introduction

Sepsis mortality rates range from 25–30%, and the incidence of sepsis is increasing in the aging population [1,2]. It is of paramount importance to advance clinical approaches for the diagnosis and treatment of the disease. The global effects of sepsis impact numerous bodily systems, including the cardiovascular, endocrine, and immune, but recently, there has been growing attention paid to the effects of sepsis on metabolism [3,4]. By definition, sepsis is a dysregulated host response to a pathogen that leads to life-threatening organ dysfunction [1]. The causative mechanism underlying organ dysfunction is likely multifaceted, but recent reports suggest mitochondrial dysfunction plays a major role [5]. Understanding mitochondrial function in sepsis represents an opportunity to develop novel and targeted therapeutics, but the assessment of mitochondrial function in humans is challenging, and not always attainable in critically ill patients. As such, new diagnostic approaches to the assessment of mitochondrial dysfunction are needed.

Mitochondrial function is a term that broadly encompasses different aspects, such as respiration rates, metabolite synthesis, calcium regulation, and membrane potential [6]. Some of these can be measured using isolated platelets, which offer a potential solution to the problem of evaluating mitochondrial function using tissue biopsies in the sepsis population. Altered platelet mitochondrial function has been observed in numerous human disease states, including type II diabetes [7], aging [8], asthma [9], and sepsis [10,11,12,13,14,15,16]. The results of the sepsis studies generally show an increase in mitochondrial activity. Although this is not a consistent finding, which warrants further study on this topic. Critically, platelet mitochondrial respiration, or mitochondrial oxygen consumption rates (mOCR), has been found to be associated with sepsis clinical parameters, such as severity of illness, organ failure, and mortality [11,12]. Metabolomics, the measurement of small molecules (<1500 Daltons), or metabolites, in a single biological sample, is an another effective approach for the study of the impact of sepsis on mitochondrial function [17]. Recently, Chacko et al., showed that the platelet metabolome in healthy subjects is functionally integrated with platelet mOCR [18]. These advancements suggest the assessment of metabolites may provide a reliable surrogate measurement of platelet mitochondrial function in a manner more suitable to human clinical trials, thus bridging the translational research gap.

To our knowledge, the evaluation of the relationship between whole blood (WB) metabolomics, platelet metabolomics, and platelet mitochondrial bioenergetics has not been previously reported. This work is needed to determine the extent of the association between platelet mOCR and metabolites identified and quantified in isolated platelets and WB. The primary aim of the study was to test the hypothesis that variations in platelet mitochondrial function, as measured by mOCR, are associated with WB and isolated platelet metabolites in sepsis patients. As a secondary aim, we sought to evaluate our methods of platelet isolation using quantitative ^1^H-nuclear magnetic resonance (NMR) metabolomics analysis by identifying the correlations between platelet and WB metabolites. Using multiple linear regression (MLR) models, we identified groups of metabolites in WB (leucine, acetylcarnitine) and platelets (creatine, ADP, glucose, taurine) that are associated with platelet mOCR.

## 2. Results

### 2.1. Patient Demographics

Thirty-one patients with sepsis and 14 control subjects were enrolled. Of the 31 sepsis subjects, 17 had mOCR matched to WB NMR metabolomics, while 14 had completed mOCR matched to platelet NMR metabolomics. Viable metabolomics data were obtained from nine of the control subjects, due to technical errors with the cell counter that precluded adjustment of metabolite concentrations to platelet count. The patient demographics and clinical parameters of the sepsis patients and control subjects are presented in Table 1, separated by regression analysis group. The sepsis cohort, when grouped by inclusion in WB or platelet regression analyses, were moderately ill sepsis patients with median sequential organ failure assessment (SOFA) scores of 5 (IQR 3, 9), and 4.5 (IQR 3, 8.5), with 28-day mortality rates of 12% and 7%, respectively.

### 2.2. Multiple Linear Regression Models Analysis

The descriptive statistics for Basal, State 4o, and Max respiration rates of the sepsis patients are presented in Table 2. We identified 31 WB and 19 platelet metabolites in at least 70% of samples (the list and concentrations of detected WB and platelet metabolites used for the analysis can be found in the excel file of the Supplementary Materials (S2.2.1); the entire data set can be found at the NIH Metabolomics Workbench: https://www.metabolomicsworkbench.org/doi:10.21228/M8FX1M). Additionally, missing values for 12 WB and 13 platelet metabolites were imputed with half of the lowest measured concentration value of each respective metabolite. A representative NMR spectrum of WB and platelet samples can be found in the Supplementary Materials (Figure S1).

The results of the MLR models relating metabolite concentrations (independent variable) to each mOCR (dependent variable) are presented in Table 3. Models using platelet metabolites were more highly associated with the platelet mOCR than those from the WB metabolites, as shown by the differences in adjusted-R^2^ and the ANOVA results. Predicted-R^2^ values followed the same hierarchy between platelet and WB models, but also revealed that the model with State 4o and WB metabolites was overfit, as evidenced by the substantial difference between the adjusted-R^2^ and the negative predicted-R^2^ (−0.111). Models meeting our criteria of significant ANOVA result and a positive predicted-R^2^ are shown as the top three models in Table 3. The stepwise models demonstrated the best relationship between State 4o and metabolites, a weaker relationship between Basal and metabolites, and no statistically significant relationships involving Max respiration.

Significant metabolites selected for inclusion in the final model were entered into MetaboAnalyst for a pathway analysis. However, due to the small number of significant compounds, results were not interpretable. Rather, we reviewed the literature surrounding the biological relevance of the metabolites in the context of sepsis. A literature review by Eckerle et al., reported statistically significant metabolites that were found to be related to three pathways, namely, energy metabolism, mitochondrial dysfunction, and platelet activation and/or aggregation (Table 4) [17].

### 2.3. Evaluation of Platelet Isolation and Metabolite Detection Methods

**Figure 1:**
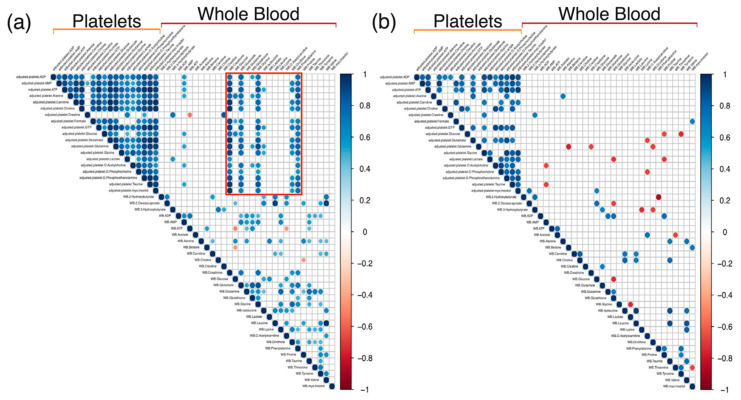
Significant associations were found between whole blood and platelet metabolites. In sepsis patients, (**a**) several whole blood metabolites (creatinine, glutamate, glycine, ornithine, phenylalanine) were significantly and positively correlated with at least 12/19 (63%) of the detected platelet metabolites; these are contained within the red box. These significant correlations were not present in the (**b**) control subjects. *p*-values of ≤0.05 were considered significant. Matrices were generated in RStudio using Pearson’s correlation coefficient (r) with a scale of −1 to 1.

To evaluate the relationship between platelet and WB metabolites in states of both sepsis and non-sepsis acute illness, we created two correlation matrices of platelet metabolites and WB metabolites in the sepsis and control subjects, respectively, which are reported in Figure 1. There were several significant correlations present in the sepsis group that were not observed in the controls, further emphasizing the metabolic perturbations provoked by the disease.

## 3. Discussion

This study provides evidence supporting the use of either platelet or WB metabolites to indirectly estimate platelet mOCR in patients with sepsis. As anticipated, platelet metabolites more accurately reflect platelet mOCR than WB metabolites, and the metabolite associations between the biofluids are more pronounced in sepsis-patients than in controls. Our data illustrate that certain WB (leucine, acetylcarnitine) and platelet (creatine, ADP, glucose, taurine) metabolites are significantly associated with platelet mOCR, as measured by high-resolution respirometry. This demonstrates that an informative, non-invasive approach to the assessment of mitochondrial function could be employed when it is not feasible to acquire tissue or platelet mitochondria for mOCR measurements in real-time, such as in the context of human clinical investigations.

Groups of WB metabolites showed a significant relationship to the Basal (unstimulated) mitochondrial respiration measurement when using a stepwise forward-backward variable selection MLR model building method. Stronger statistical models were achieved with platelet metabolites as potential predictors (adj-R^2^: 0.836, 0.711 vs. adj-R^2^: 0.428) with the same model building method. This is logical because platelet mitochondria likely contribute substantially to the components of the platelet metabolome. Moreover, the difference in model strength might be explained by the relatively lower biomass of platelets compared to other tissues. Of note, the associations between metabolites with Basal and State 4o respiration differ. This is expected, as Basal respiration represents the passive energy production state, while State 4o is a non-physiologic state, which indirectly measures the leakage of protons across the inner mitochondrial membrane. Increased levels of State 4o may represent ROS-associated damage to the inner mitochondrial membrane, and increased uncoupling protein leading to reduced energy output [19,20,21]. This suggests it may be possible to use different metabolites (or metabolite panels) to probe various mitochondrial processes.

Three models met the criteria of significant ANOVA results and positive predicted-R^2^ values. The metabolites identified via these models appear to have biological significance with sepsis and the prespecified goal of the project, namely, development of non-invasive reflections of altered mitochondrial function, adding to the clinical relevance of these findings (Table 4).

In regard to specific metabolites and pathways, it is worth noting that both of the platelet models included ADP, which highlights the importance of this molecule in sepsis. ADP and creatine are both substrates of ATP synthesis, but ADP is also maintained in high concentrations within the dense granules of platelets as they utilize the compound to activate aggregation [22,23,24]. Platelet aggregation is also strongly influenced by choline, which is a precursor to platelet activating factor [25]. Sepsis is characterized as a hypercoagulable state, so it is logical that ADP and choline are highlighted in this biological and clinical model of sepsis.

Other metabolic signals were also evident. Glucose metabolism in sepsis has been extensively studied. In the context of activated platelets, there is evidence to support increases in glucose uptake, glycolysis, lactate, and glycogenolysis [26]. Notably, the role of taurine in sepsis is unclear; but it is highly concentrated in platelets and reportedly reduces coagulation and increases during animal hibernation [27,28]. We find the latter observation interesting, because it may play a role in the sepsis hibernation hypothesis, which theorizes that downregulated mitochondrial respiration acts as a defensive strategy to prevent sepsis-induced cell death [29].

Lastly, acetylcarnitine and leucine have ties to sepsis through mitochondrial dysfunction and energy metabolism, respectively. Leucine is a branched chain amino acid, which are used as a source of energy during sepsis [30]. Recently, Puskarich et al., and Chung et al., both found acetylcarnitine to be associated with mortality and organ failure in sepsis [31,32]. These findings, in conjunction with ours, further support the previously observed relationship between acetylcarnitine, platelet mitochondrial respiration, mortality, and organ failure [12]. Additional studies are needed to identify the mechanisms that underpin these associations, but these biological relationships support the utility of a metabolomics approach to estimate platelet mitochondrial function.

Despite the well-characterized metabolic derangement of sepsis, there is a major inability to accurately and longitudinally assess mitochondrial function in these patients, due to the ethical and practical concerns of obtaining muscle/tissue biopsies. As such, there remains a clinical and critical need to non-invasively evaluate sepsis-induced changes in mitochondrial function for several reasons. First, mitochondrial function has been identified as increasingly important in contributing to multiorgan dysfunction [5,33]. Second, a reliable and practical method could be more easily translated into clinical use to identify metabolically impaired phenotypes. There is a growing body of literature to support the use of measuring mitochondrial function in peripheral blood cells from patients with sepsis [10,11,12,13,14,15,16]. The clinical significance of platelet mitochondrial function is evident in recent reports that show associations between increased respiration with mortality and organ failure in sepsis patients [11,12]. To our knowledge, this is the first study to relate metabolomics data from two distinct biofluids with platelet mitochondrial bioenergetics. These results are consistent with other studies using intact platelets isolated from human blood samples. The high resolution respirometry used in these studies is generally considered the gold standard for measuring mitochondrial oxygen consumption. However, this experimental technique has several pitfalls that may prevent its translation into the clinical toolbox. The time sensitive method requires skilled technicians to collect fresh blood samples, isolate platelets, and execute the respirometer protocol without the ability to freeze for later use or reruns. As such, this is not a high throughout procedure. Therefore, the purpose of this study was to discover and evaluate sets of metabolites that reflect platelet mOCR, potentially bypassing the need for high-resolution respirometry in clinical settings. We recognize our hypothesis-generating results only lay the groundwork that must be refined and validated in larger cohorts and more generalizable populations, but the future directions seem promising.

The stronger association between the platelet mOCR and platelet metabolome supports the secondary goal of evaluating our platelet isolation methods using quantitative ^1^H-NMR spectroscopy. The apparent differences in detected metabolites and significant correlations between the platelet and WB metabolomes supports the successful optimization of our platelet metabolomics approach. Indeed, the appearance of associations between the WB and platelet metabolomes in the subjects with sepsis emphasizes the prolific metabolic response that sepsis provokes. Platelets release metabolites into the blood upon activation, which offers a potential mechanistic explanation for this observation [22,23,24]. Albeit, there were fewer control subjects than in the sepsis group, so this may affect the lack of associations between the platelet and WB metabolites in this group.

We recognize there are several limitations of our study. First and foremost, this is a small, single-center sample, and the specific metabolites and panels should be viewed skeptically and reproduced prior to broader application. Just as important, the associations pursued were matched to platelets, which represent a small biomass that may or may not reflect vital organ mitochondrial function. Future studies should attempt to make this pathophysiologic link. It is worth noting that the maximum respiration models were consistently weaker than those of the other two mOCR. The lack of significant relationships with Max respiration and measured metabolites is reasonable, as this represents an artificial experimental state of maximum respiratory capacity that is not reflected in the body, and would not necessarily be expected to be related to measured peripheral metabolites. In regards to patient outcomes, we did not reproduce the previously reported association between maximum respiratory rate and SOFA score [12], although this may reflect our small sample size and relatively low and homogeneous severity of illness compared to prior reports. Again, due to a relatively lower severity of illness, we did not observe significant differences in respiratory rates and mortality as previously reported [11,12]. When building an MLR model with such interrelated variables like metabolites, we considered the possibility of collinearity between them. However, this is not of concern, as the intention of our model was to use metabolite sets to estimate platelet mOCR and not individual metabolites. We refrained from interpreting the β Coefficients from the MLR models to avoid over-speculation about the relationships between individual metabolites and mitochondrial respiration. The ANOVA tested the difference between the selected models and the saturated models that includes every measured metabolite for that biofluid. The full model has more predictors than the sample size, which puts the test at risk of overfitting. However, the resulting *p*-values were not excessively small, so we do not believe the ANOVA tests were overfit. Although, due to the small sample size, the test result could have been idiosyncratic to our population, leading to the need for external validation. Ideally, we would have compared the models from sepsis patients and non-sepsis controls, however, the mOCR data from the controls was considered unusable, due to technician errors that occurred during experimentation. Albeit, this limitation did not prevent us from achieving the goal of the study, which was to determine if metabolomics data are reflective of platelet mitochondrial respiration. A follow-up study is necessary to identify potential differences and clinical significance between the metabolic associations in sepsis and non-sepsis subjects. The cross-sectional design of the study was also a limitation that precluded us from evaluating metabolic changes over time.

## 4. Materials and Methods

### 4.1. Setting

This was a prospective observational study of patients that were treated with an institutional quantitative resuscitation protocol for sepsis in the emergency department of a university medical center; a separate cohort of subjects were enrolled for the purpose of acquiring WB and platelet samples to serve as negative, non-sepsis controls. The protocol was IRB-approved (IRB#2016-0076) at the University of Mississippi Medical Center. All patients or their legally authorized representative (sepsis patients) provided written informed consent.

### 4.2. Participants

The enrollment period took place from 09/2016–9/2018. Enrollment criteria were the following: (1) Suspected or confirmed infection; (2) Any two of four criteria of systemic inflammatory response in ED [34]; (3) Age ≥ 18; (4) Lactate ≥ 2.0 mmol/L; (5) Enrollment within 2 h of initiation of quantitative resuscitation protocol [35]. Exclusion criteria were: (1) any primary diagnosis other than sepsis; (2) established Do Not Resuscitate status; (3) transferred from another hospital with sepsis therapy already initiated; (4) cardiopulmonary resuscitation (chest compression or defibrillation) prior to enrollment; (5) patient or legal representative unable to understand and sign informed consent.

Controls patients were eligible if they were admitted to the emergency department and had no medical conditions that required chronic administration of medication expected to affect platelet function (aspirin, PGY12 inhibitors, etc.). Unlike the sepsis cohort, these patients were not admitted to the intensive care unit. Controls were attempted to be matched to sepsis patients in a 1:1 fashion by sex, race, and age ± 10 years. Not all patients enrolled had an eligible control match enrolled. This control group was chosen to represent an acutely ill cohort of the same race, sex, and age as the sepsis cohort, so that differences in the group were more likely to be attributed to sepsis, rather than general acute illness or differences in baseline demographics known to impact mitochondrial function [36].

### 4.3. Collection of Blood Samples

After discarding a waste of 3–5 mL, three WB samples (12 mL each) were collected by direct venipuncture or from an indwelling line into K2 EDTA-containing Vacutainer tubes (BD 367863; Becton-Dickinson, Franklin Lakes, NJ, USA); each tube was inverted 6 to 8 times to ensure distribution of the anti-coagulant. Samples were immediately placed on ice and promptly transported to the laboratory. The blood sample for WB metabolomics was aliquoted into screw-top cryotubes (1 mL), flash-frozen in liquid nitrogen and immediately stored (−80 °C). An aliquot of WB from each subject was shipped frozen on dry ice to the University of Michigan’s NMR Metabolomics Laboratory.

### 4.4. Platelet Isolation for Assessment of Mitochondrial Respiration

Platelet isolation for measurement of mOCR was performed as previously described with minor modifications [12]. Briefly, blood (12 mL) was centrifuged (200× g for 6 min at room temperature). The platelet-rich plasma layer was transferred to another tube and centrifuged (4500× *g* for 5 min at 4 °C) to pellet the platelets. The nearly cell-free plasma layer was transferred to a new tube, leaving approximately 0.25 mL of plasma in the original tube. This remaining plasma was used to resuspend the platelet pellet to produce ultra-rich plasma. Platelets in the ultra-rich plasma were counted with an automated cell counter (Cellometer AutoM10; Nexcelom Bioscience, Lawrence, MA, USA) and diluted with additional plasma as needed to achieve a concentration of ~200 × 10^6^ cells/mL. This cell concentration has previously been shown to be optimal for mitochondrial respiration assessment [11]. Measurements were performed in the patient’s own plasma instead of buffer media consistent with prior work by our group [12], as mOCR appear to be influenced by factors in the plasma [37].

### 4.5. Sample Extraction for Metabolomics

Platelets were isolated and counted as described above. In preparation for metabolomics assay, resuspended platelets were centrifuged (4500× g, 5 min, at 4 °C), decanted, and then resuspended in 1 mL of methanol (20 °C). Cell lysis was achieved by flash-freezing samples in liquid nitrogen for 30 s and allowing them to thaw to room temperature, before storage at −80 °C. Frozen samples were shipped on dry ice to the University of Michigan’s NMR Metabolomics Laboratory for analysis, where they were stored at −80 °C. Immediately before assay, samples underwent a second freeze-thaw cycle by flash-freezing in liquid nitrogen and thawing to room temperature [38]. The researchers at the NMR Metabolomics Laboratory were blinded to the experimental arms.

Platelet pellets were on ice for the duration of the extraction. Samples were transferred to 5-mL centrifuge tubes, and chloroform was added to each resuspended pellet to create a 1:1 methanol:chloroform solution. An additional 250 uL of 1:1 methanol:chloroform was added, then 1 mL DI water, followed by a final 500 uL addition of DI water. After each solvent addition, samples were vortexed (30 s). After the final addition of water, samples were vortexed until they were white and opaque. Samples were chilled in ice-water bath (15 min), then centrifuged (1000× g, 15 min, at 4 °C). After centrifugation, a thin pellet of cellular debris and precipitated protein separated the upper aqueous layer of the extracted sample from the lower chloroform layer. The aqueous supernatant was removed, lyophilized, and resuspended in 50 mM phosphate buffer in deuterium oxide in preparation for NMR. WB samples were prepared for NMR by methanol:chloroform precipitation as previously described [39].

### 4.6. Platelet Mitochondrial Respiration Measurements

Mitochondrial oxygen consumption was measured using a high-resolution respirometer (Oxygraph O2k; Oroboros Instruments, Innsbruck, Austria), by a technician that was aware of the experimental groups. The device was calibrated and the data were acquired in accordance with the manufacturer’s instructions, as previously reported [12]. The platelet concentrations in the chamber were entered into the manufacturer-provided software (DatLab 5.2; Oroboros Instruments, Innsbruck, Austria), which allowed for normalization of the results to cell count at the end of the experiment.

As previously described, each mOCR was measured in the following order; Basal, State 4o, maximum (Max) respiration [12]. Briefly, unstimulated platelets provided the resting Basal respiration rate of oxidative phosphorylation. Following the baseline measurement, oligomycin (3 µL; 4 µg/mL from 95% HPLC pure oligomycin A) was added to prevent ATP production by inhibiting ATP synthase (complex V), representing the oxygen consumed by proton leakage across the inner mitochondrial membrane (State 4o). Then, sequential additions (2 µL, then 1 µL) of carbon cyanide 4-(trifluoromethoxy) phenylhydrazone (FCCP; 20 Mm—yellow) were added to measure Max respiration [19]. Our Max rate represents the maximum respiration when using an intact cell model such as this, however, it does not represent the same maximum rate as determined when using isolated mitochondria in the presence of excess substrates [6]. It is important to note that these measurements included oxygen consumption of the plasma, along with other extra-mitochondrial activity. In order to account for these contributions, rotenone (3 µL) and antimycin A (3 µL) were added at the end of the assay to achieve final concentrations of 0.6 μmol/L and 1.8 mmol/L, respectively, to measure the residual OCR that is independent of mitochondrial oxygen consumption; this value was subtracted from each mOCR measurement. The selected respiration rates have been published in previous studies of platelet mitochondrial function in sepsis patients [11,12]. These mOCR are measured in intact platelets, rather than permeabilized cells or isolated mitochondria. This minimizes the cellular disruptions inflicted by the isolation or permeabilization process, a critical step when using reactive clinical samples such as platelets from sepsis patients. We suspended the platelets in each patient’s own plasma to further replicate the in vivo environment. All chemicals for the platelet mitochondrial experiments were purchased from Sigma-Aldrich (St. Louis, MO, USA).

### 4.7. Quantitative 1-D- ^1^H-NMR Metabolomics

At the time of assay, samples were thawed on ice and prepared for NMR analysis, as previously described [39]. Details of NMR acquisition can be found in the Supplementary Materials (S4.7.1). NMR spectra were acquired at the University of Michigan BioNMR Core Laboratory on a Bruker 18.8 Tesla (800 MHz) NMR spectrometer ascend magnet, equipped with a 5 mm Triple resonance inverse detection TCI cryoprobe, and Bruker NEO console, operated by TopSpin 4.0.7 software. Details of NMR spectra acquisition, the pulse sequence, and spectral analysis can be found in the Supplementary Materials (S4.7.1). Resulting metabolomics data were scaled to correct for differences in initial sample volume (for WB samples) or cell count (isolated platelets) before statistical analysis.

### 4.8. Data Analysis

Missing values were imputed using half of the minimum value of each respective metabolite concentration. The metabolomics data were then normalized by natural-log transformation. Descriptive statistics were used to summarize patient demographic data. All statistical analyses were performed, and all correlation matrices were constructed in RStudio (RStudio Team 2015. RStudio: Integrated Development for R. RStudio, Inc., Boston, MA, USA). The primary associations were done using each mOCR as the dependent (response, y) variable and the natural-log transformed metabolite concentrations as the independent (predictors, x) variables for a MLR model. Each MLR model was built using the stepwise forward-backward variable selection method when given the respective metabolites. The metabolites were entered or removed from the model based on the default *p*-values, 0.1 and 0.3, respectively. Adjusted-R^2^ shows the goodness-of-fit of the model to the data and predicted-R^2^ protects against overfitting the model. The R code for the MLR models is provided in the Supplementary Materials (S4.8.1).

To test significance of the models, ANOVA was used to compare each final model to the full model, which included all possible metabolites as predictors. Resulting *p*-values of ≤ 0.05 were considered significant. MetaboAnalyst 4.0 (https://www.metaboanalyst.ca/) was used to conduct a pathway analysis of the metabolites from the final MLR models [40]. Pearson’s correlation was utilized to test the associations between the platelet and WB metabolites.

## 5. Conclusions

In conclusion, WB (leucine, acetylcarnitine) and platelet (creatine, ADP, glucose, taurine) metabolites have potential use as surrogates for estimating platelet mOCR. This strategy, which circumvents the need for tissue sampling and the use of high-resolution respirometry, could be a viable approach to assess mitochondrial function in patients with sepsis. Further studies are warranted to corroborate these findings in a larger, more generalizable cohort.

## Figures and Tables

**Table 1:** Patient demographics and clinical parameters.

Variable	Sepsis Patients		Controls (n = 9)
	Whole Blood (n = 17)	Platelet (n = 14)	
Age (IQR) *	59 (52–67)	57 (50–67)	53 (32–56)
Race (%)			
White	8 (47)	8 (57)	2 (22)
African-American	9 (53)	6 (43)	7 (78)
Ethnicity (%)			
Non-Hispanic	17 (100)	14 (100)	9 (100)
Hispanic	0 (0)	0 (0)	0 (0)
Sex (%)			
Male	10 (59)	8 (57)	4 (44)
Female	7 (41)	6 (43)	5 (56)
28-day mortality (%)	2 (12)	1 (7)	0 (0)
BMI kg/m^2^ (IQR) *	25.6 (23–34)	26.6 (23–33)	31.5 (24–46)
Preexisting conditions (%)			
Coronary artery disease	2 (12)	2 (14)	0 (0)
End-stage renal disease	2 (12)	2 (14)	0 (0)
Chronic obstructive pulmonary disease	5 (29)	5 (36)	1 (11)
Chronic heart failure	0 (0)	0 (0)	1 (11)
Cirrhosis	1 (6)	0 (0)	0 (0)
Peripheral vascular disease	1 (6)	1 (7)	0 (0)
Cerebrovascular accident	1 (6)	2 (14)	0 (0)
Malignancy	4 (24)	1 (7)	0 (0)
Vital signs (IQR) *			
Heart rate (beats/min)	102 (86–106)	102 (87–107)	85 (77–88)
Systolic blood pressure (mmHg)	110 (104–119)	110 (98–116)	145 (128–149)
Diastolic blood pressure (mmHg)	65 (59–72)	64 (59–68)	84 (81–98)
Baseline laboratory (SD)			
Creatinine (mg/dL)	1.8 (1.3)	1.6 (1.2)	n/a
Platelet count (×1000 cells/mm^3^)	205 (148)	182 (100)	n/a
White blood count (×1000 cells/mm^3^)	15 (7.8)	15.75 (8.2)	n/a
Disease severity (IQR) *			
SOFA (enrollment)	5 (3–9)	4.5 (3–8.5)	n/a
Lactate mM (enrollment)	1.8 (1.2–2.1)	2 (1.2–2.1)	n/a

* Median (interquartile range); n/a = not applicable.

**Table 2:** Mitochondrial Oxygen Consumption Descriptive Statistics.

Statistic	Basal	State 4o	Max
Median	0.0861	0.0191	0.1141
IQR	0.0626–0.1117	0.0060–0.0267	0.1010–0.1364

IQR = interquartile range; all units are pmol/(sec * 10^6^ platelets).

**Table 3:** Multiple Linear Regression results.

Response (y)	Covariates (x)	β Coefficient (*p*-Value)	Adj.-R^2^	Pred.- R^2^	ANOVA (*p*-Value)
State 4o	PLT.Creatine	−0.009 (0.081)	0.836	0.629	(0.0003) *
	PLT.ADP	0.066 (0.000) *			
	PLT.Choline	−0.047 (0.000) *			
	PLT.Glucose	0.015 (0.026) *			
Basal	PLT.ADP	0.141 (0.000) *	0.711	0.608	(0.0004) *
	PLT.Taurine	−0.107 (0.000) *			
Basal	WB.Leucine	0.084 (0.002) *	0.428	0.308	(0.0079) *
	WB.O.Acetylcarnitine	−0.049 (0.066)			
State 4o	WB.Alanine	0.042 (0.060)	0.281	−0.111	(0.039) *
	WB.2.Hydroxybutyrate	0.026 (0.099)			
Max	WB.3.Hydroxybutyrate	0.017 (0.049) *	0.236	−0.033	(0.0595)
	WB.AMP	0.032 (0.088)			
Max	PLT.Creatine	0.022 (0.080)	0.170	−0.147	(0.0801)

PLT.*metabolite* = platelet metabolite, WB.*metabolite* = whole blood metabolite; * indicates *p*-value ≤ 0.05.

**Table 4:** Selected Metabolites Biological Relevance to Sepsis.

Sepsis Pathway	Metabolites	
	Whole Blood	Platelet
Energy metabolism	Leucine	Creatine, ADP *, Choline *, Glucose *
Mitochondrial dysfunction	Acetylcarnitine	n/a
Platelet activation/aggregation	Taurine	ADP *, Choline *, Glucose *

* Metabolites that are related to several sepsis pathways; n/a = not applicable; [17].

## Supplementary Materials

The following are available online at https://www.mdpi.com/2218-1989/10/4/139/s1; S2.2.1 WB and platelet metabolites with mOCR data; Figure S1: Representative NMR spectra from whole blood and platelet extracts; S4.7.1 Acquisition of Quantitative 1-D-1H-NMR Metabolomics Data; S4.8.1 Stepwise forward-backward variable selection model building R code.
