# Supplementary material for: A Multivariate Metabolomics Method for Estimating Platelet Mitochondrial Oxygen Consumption Rates in Patients with Sepsis
Source: Metabolites. 2020 Apr 2;10(4):139. doi: 10.3390/metabo10040139 (PMC7240966; doi:10.3390/metabo10040139)
Supplement: Supplementary file 1 [file metabolites-10-00139-s001.zip › McCann M-platelet manuscript-supplement files/S4.7.1 Acquisition of Quantitative 1-D-1H-NMR Metabolomics Data.docx]

*S4.7.1 Acquisition of Quantitative 1-D-1H-NMR Metabolomics Data*

At the time of assay, samples were thawed on ice and prepared for NMR analysis as previously described [34]. Sample volume was measured and recorded. An internal standard, sodium 2,2-dimethyl- 2-silapentane-5-sulfonate-d6 (DSS-d_6_) of known concentration with 0.2% sodium azide (Chenomx Inc, Edmonton, AB, CA) was added to each sample. Sample pH was measured and corrected to 6.5-7.5 by dropwise addition of 0.1 mM deuterium chloride (CIL) or sodium deuteroxide (CIL). Samples were transferred to 5mm 800 MHz precision NMR tubes (Wilmad Lab Glass, Vineland, NJ, USA) for assay.

NMR spectra were acquired at the University of Michigan BioNMR Core Laboratory on a Bruker 18.8 Tesla (800 MHz) NMR spectrometer ascend magnet equipped with a 5mm Triple resonance inverse detection TCI cryoprobe and Bruker NEO console, operated by TopSpin 4.0.7 software. NMR spectra were recorded using the Bruker pulse sequence noesygppr1d. Spectra were collected with 32 scans for WB extracts, and with 128 scans for platelet extracts. The NMR pulse sequence was as follows: a 10 ms recovery, a 990 ms saturation pulse, two calibrated 90° pulses, a mixing time of 100 ms, a final 90° pulse and an acquisition period of 4 s. Receiver gain was set to 0.5 for all samples. Optimal excitation pulse widths were obtained by utilizing an array of pulse lengths as previously described (1).

NMR spectra of platelets and WB were analyzed with Chenomx NMR Suite 8.2 (Edmonton, AB, Canada) software. The Processor module was used to phase shift, baseline correct and excise water from each spectrum as previously described (1). Compounds were then identified and quantified using the profiler module of the software, which allows metabolites to be quantified relative to an internal standard of known concentration (1). All identifiable compounds were quantified in platelet extracts. In WB samples, a list of compounds to profile in every sample was selected after profiling 16 samples, additionally, any unprofiled peaks were checked to determine if they corresponded to identifiable compounds, in which case they would be profiled. Any volatile compounds (eg. ethanol) were removed from dataset prior to analysis, as lyophilization may inconsistently remove these compounds from extracts. Resulting data were scaled to correct for differences in initial sample volume (for WB samples) or cell count (isolated platelets) before statistical analysis.
