# Supplementary material for: A Multivariate Metabolomics Method for Estimating Platelet Mitochondrial Oxygen Consumption Rates in Patients with Sepsis
Source: Metabolites. 2020 Apr 2;10(4):139. doi: 10.3390/metabo10040139 (PMC7240966; doi:10.3390/metabo10040139)
Supplement: Supplementary file 1 [file metabolites-10-00139-s001.zip › McCann M-platelet manuscript-supplement files/S4.8.1 Stepwise forward-backward variable selection model building R code.docx]

*S4.8.1 Stepwise forward-backward variable selection model building R code*

#Basal

##wb metabolites

model <- tmp$Basal.Rox~.

df <- tmp[c(24:54)]

fit <- lm(model, log(df))

ols_step_both_p(fit, pent = 0.1, prem = 0.3, details = FALSE)

##platelet metabolites

model <- tmp$Basal.Rox~.

df <- tmp[c(5:23)]

fit <- lm(model, log(df))

ols_step_both_p(fit, pent = 0.1, prem = 0.3, details = FALSE)

#State 4o

##wb metabolites

model <- tmp$State.4o.Rox~.

df <- tmp[c(24:54)]

fit <- lm(model, log(df))

ols_step_both_p(fit, pent = 0.1, prem = 0.3, details = FALSE)

##platelet metabolites

model <- tmp$State.4o.Rox~.

df <- tmp[c(5:23)]

fit <- lm(model, log(df))

ols_step_both_p(fit, pent = 0.1, prem = 0.3, details = FALSE)

#Max

##wb metabolites

model <- tmp$Max.Rox~.

df <- tmp[c(24:54)]

fit <- lm(model, log(df))

ols_step_both_p(fit, pent = 0.1, prem = 0.3, details = FALSE)

##platelet metabolites

model <- tmp$Max.Rox~.

df <- tmp[c(5:23)]

fit <- lm(model, log(df))

ols_step_both_p(fit, pent = 0.1, prem = 0.3, details = FALSE)

Correlation matrices of platelet and whole blood metabolites in controls and sepsis

#1 plt vs wb sepsis

View(tmp)

cor <- subset(tmp, select=c(5:54))

View(cor)

res2 <- rcorr(as.matrix(cor))

library(corrplot)

corrplot(res2$r, type = "upper", order = "hclust",

tl.col = "black", tl.srt = 45, tl.cex= 0.35,

bg="white", p.mat = res2$P, sig.level = 0.05, insig = "blank")

corrplot(res2$r, type = "upper",

tl.col = "black", tl.srt = 45, tl.cex= 0.35,

bg="white", p.mat = res2$P, sig.level = 0.05, insig = "blank")

#2 plt vs wb controls

tmp2 <- read.csv("impute2_controls_plt_wb.csv")

View(tmp2)

cor <- subset(tmp2, select=c(33:51,2:32))

View(cor)

res2 <- rcorr(as.matrix(cor))

library(corrplot)

corrplot(res2$r, type = "upper", order = "hclust",

tl.col = "black", tl.srt = 45, tl.cex= 0.35,

bg="white", p.mat = res2$P, sig.level = 0.05, insig = "blank")

corrplot(res2$r, type = "upper",

tl.col = "black", tl.srt = 45, tl.cex= 0.35,

bg="white", p.mat = res2$P, sig.level = 0.05, insig = "blank")
