# Supplementary material for: A Multivariate Metabolomics Method for Estimating Platelet Mitochondrial Oxygen Consumption Rates in Patients with Sepsis
Source: Metabolites. 2020 Apr 2;10(4):139. doi: 10.3390/metabo10040139 (PMC7240966; doi:10.3390/metabo10040139)
Supplement: Supplementary file 1 [file metabolites-10-00139-s001.zip › McCann M-platelet manuscript-supplement files/Figure S1 Representative NMR spectra from whole blood and platelet extracts..docx]

*Figure S1 Representative NMR spectra from whole blood and platelet extracts.*

**Figure S1** Spectra were acquired and analyzed as described in SDC 1. Insets illustrate selected metabolite peaks that were significant in (**A**) whole blood (leucine) and (**B**) platelet (taurine) samples. Shaded regions of chemical structures highlight the protons detected by inset peaks. Methanol is evident in (**A**) whole blood secondary to the extraction process.
